# Low Hemoglobin A1c (HbA1c) Revealing Hemolytic Anemia in a Growth Hormone-Treated Child: A Case Report

**DOI:** 10.7759/cureus.91386

**Published:** 2025-09-01

**Authors:** Tomoyuki Ito, Yoichiro Oda, Shota Kato, Takahiro Shindo, Yoshiyuki Namai

**Affiliations:** 1 Department of Pediatrics, Ohta General Hospital Foundation, Ohta Nishinouchi Hospital, Koriyama, JPN; 2 Department of Pediatrics, Chigasaki Municipal Hospital, Chigasaki, JPN; 3 Department of Pediatrics, Graduate School of Medicine, The University of Tokyo, Tokyo, JPN

**Keywords:** diabetes mellitus, glycoalbumin, growth hormone deficiency, hemoglobin a1c, hemolytic anemia, hereditary spherocytosis, osmotic fragility test

## Abstract

Hemoglobin A1c (HbA1c) is widely utilized for monitoring glycemic control in diabetes mellitus. Growth hormone (GH) therapy necessitates regular glucose monitoring due to an increased risk of diabetes. However, hemolytic anemia may result in spuriously low HbA1c levels due to a shortened erythrocyte lifespan, potentially leading to misinterpretation of glycemic status. We report an 11-year-old girl with GH deficiency undergoing GH therapy who presented with an abnormally low HbA1c level of 3.9% despite normal glucose tolerance. Laboratory tests revealed mild anemia (hemoglobin: 11.8 g/dL), elevated total bilirubin (1.27 mg/dL), and undetectable haptoglobin (<10 mg/dL), suggesting ongoing hemolysis. Osmotic fragility testing revealed erythrocyte sensitivity to hemolysis, which was more pronounced in incubated blood than in fresh blood. Although a definitive diagnosis remained elusive owing to the lack of Coombs testing and genetic analysis, hereditary spherocytosis (HS) was considered the most likely etiology, particularly given the epidemiological prevalence of congenital hemolytic disorders in Japan. Due to unreliable HbA1c values, glycoalbumin was used as an alternative marker for glucose monitoring, demonstrating normal levels (13.7%). This case highlights the need to consider hemolytic anemia in pediatric patients undergoing GH therapy who present with unexpectedly low HbA1c values. In such cases, glycoalbumin may serve as a reliable alternative marker for assessing glucose metabolism.

## Introduction

Hemoglobin A1c (HbA1c) is widely utilized as an indicator for monitoring glycemic control in the diagnosis and management of diabetes mellitus (DM) [1]. This measurement reflects the amount of glycated hemoglobin accumulated over the lifespan of erythrocytes. The glycation of hemoglobin occurs through a non-enzymatic reaction between circulating glucose and the N-terminal valine of the hemoglobin β-chain. This reaction initially forms a labile Schiff base, which subsequently undergoes an Amadori rearrangement to yield a stable ketoamine product [2]. HbA1c is considered a reliable indicator of long-term glycemic control due to its resistance to daily fluctuations in blood glucose levels and its relative stability during acute illnesses or stress-induced glycemic changes [3]. Growth hormone (GH) therapy is associated with an increased incidence of type 2 DM [4], necessitating periodic monitoring. In hemolytic anemia, the shortened lifespan of erythrocytes results in spuriously low HbA1c levels [5]. In the present patient, hemolytic anemia was considered following the detection of an abnormally low HbA1c level during GH therapy follow-up.

## Case presentation

An 11-year-old girl was diagnosed with GH deficiency at the age of 10 years based on an endocrinological evaluation, which revealed peak GH levels of 3.46 and 1.07 ng/mL in response to clonidine and levodopa loading, respectively. GH therapy was subsequently initiated. She had no history of neonatal jaundice or parvovirus infection. Subsequently, she was referred to our hospital following a change in residence. Routine blood tests revealed an abnormally low HbA1c level of 3.9% (measured by high-performance liquid chromatography (HPLC) method). To investigate the underlying cause, further blood tests were performed (Table 1). Concordant HbA1c values were confirmed using HPLC and immunoassay methods. Laboratory findings indicated mild anemia (hemoglobin: 11.8 g/dL; reference range, 11.9-14.9 g/dL), mildly elevated total bilirubin levels (1.27 mg/dL; reference range, 0.25-1.10 mg/dL), and undetectable haptoglobin (<10 mg/dL), suggestive of ongoing hemolysis.

**Table 1:** Laboratory results

Item			Reference range
Complete blood cell count			
White blood cell count	10800	/μL	3800-10100
Hemoglobin	11.8	g/dL	11.9-14.9
Mean corpuscular volume	83.2	fL	78.0-93.0
Mean corpuscular hemoglobin concentration	36.2	%	32.5-36.0
Platelet count	35.1	× 10^4^/μL	18.0-44.0
Reticulocyte	55.3	‰	3-22
Biochemistry			
Glucose	84	mg/dL	73-109
Hemoglobin A1c (high-performance liquid chromatography)	3.9	%	4.9-6.0
Hemoglobin A1c (immunoassay)	3.7	%	4.6-6.2
Albumin	4.6	g/dL	3.8-4.7
Aspartate aminotransferase	20	U/L	15-30
Alanine aminotransferase	9	U/L	9-28
Lactate dehydrogenase	215	U/L	145-270
Total bilirubin	1.27	mg/dL	0.3-1.1
Indirect bilirubin	0.93	mg/dL	0.2-1.0
Blood urea nitrogen	13.1	mg/dL	6.8-19.2
Creatinine	0.43	mg/dL	0.39-0.69
Sodium	139	mEq/L	138-144
Potassium	4.1	mEq/L	3.6-4.7
Chloride	103	mEq/L	102-109
Calcium	9.5	mg/dL	8.7-10.1
Iron	56	μg/dL	40-188
Unsaturated iron-binding capacity	217	μg/dL	155-350
Total iron-binding capacity	273	μg/dL	250-410
Ferritin	94.9	ng/mL	4.63-204
Haptoglobin	<10	mg/dL	19-170

**Figure 1:**
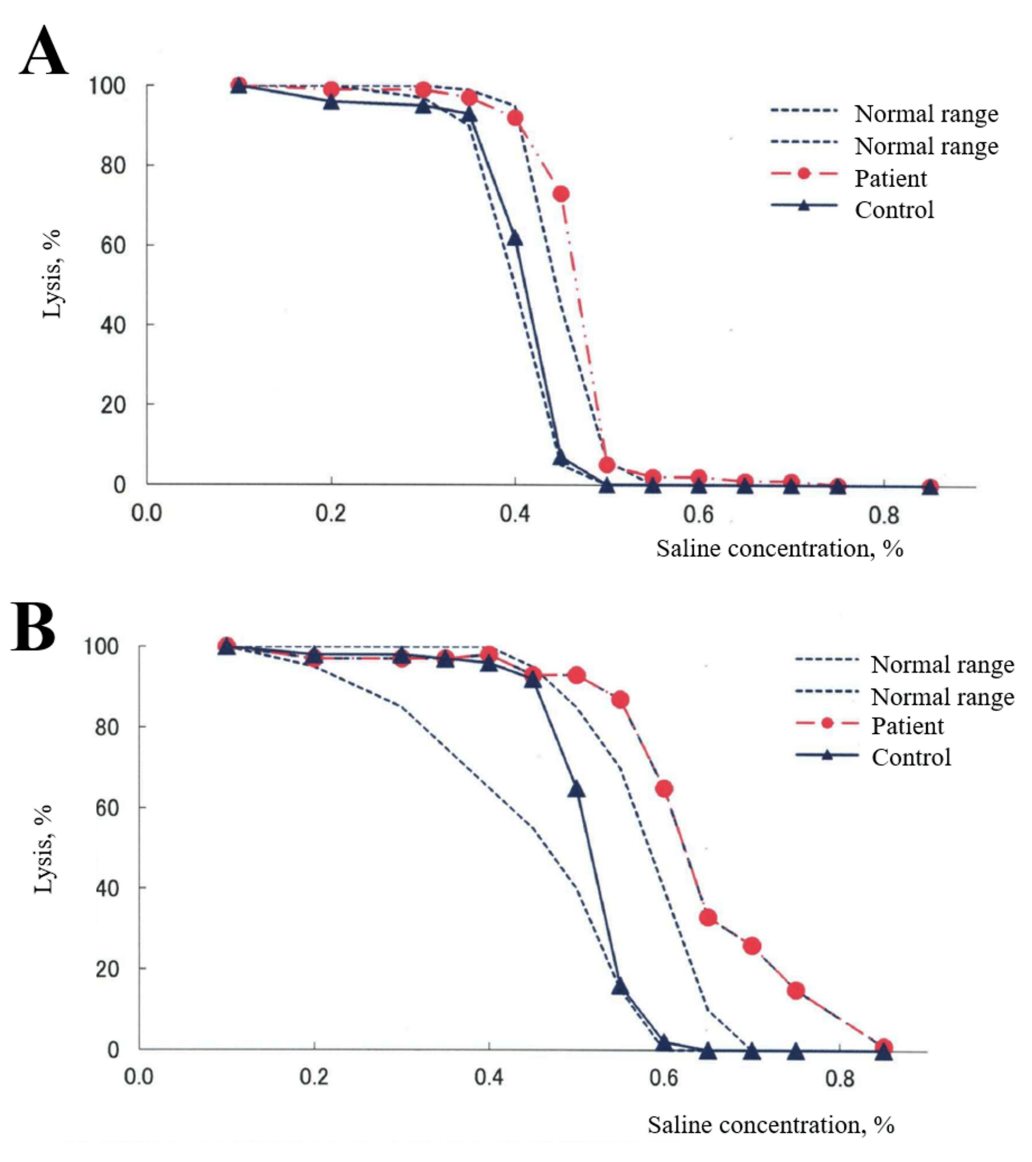
Osmotic fragility test for a fresh blood sample (A) and a blood sample with a 24-hour incubation (B) The dashed lines depict the normal range.

A family history revealed anemia in the patient’s mother and maternal grandmother, whereas her brother exhibited no hematological abnormalities. No other significant familial medical history was noted. Although the peripheral blood smear analysis revealed no morphological irregularities, the osmotic fragility test demonstrated increased erythrocyte sensitivity to hemolysis, particularly in incubated blood samples compared with fresh blood (Figure 1). Although hereditary spherocytosis (HS) or other red blood cell membrane disorders were suspected, further genetic testing was not pursued, thereby precluding a definitive diagnosis.

Due to the challenges in accurately assessing HbA1c, glycoalbumin levels were measured and found to be within the normal range (13.7%; reference range: 12.3%-16.5%). Consequently, glycoalbumin was adopted as the primary marker for future evaluation of glucose tolerance.

Informed consent for the publication of this case report was obtained from the patient’s legal guardians.

## Discussion

In the present patient, a hemolytic disease was clinically suspected based on markedly reduced HbA1c levels and abnormal erythrocyte osmotic fragility test results. Although a definitive diagnosis remained elusive due to the absence of a Coombs test and genetic analysis, HS was considered the most likely etiology, particularly given its relatively high prevalence among congenital hemolytic disorders in Japan [6]. This clinical suspicion is further supported by reports indicating that approximately 20% of patients with HS do not exhibit characteristic spherocytes on peripheral blood smears [7]. A comprehensive literature search was conducted on PubMed, BMJ Journals, and Cases Database to investigate potential associations between hemolytic anemia and the following parameters: GH, hemoglobin A1c, and HbA1c. No direct relationships were identified in the current literature. Moreover, to the best of our knowledge, no previous case reports have documented the diagnosis of hemolytic anemia through HbA1c screening during GH therapy.

The Action to Control Cardiovascular Risk in Diabetes (ACCORD) trial demonstrated that intensive glycemic control targeting an HbA1c level below 6.0% (42 mmol/mol) led to increased mortality and a higher incidence of hypoglycemia, particularly in patients with a high observed-to-predicted HbA1c ratio [8]. These findings highlighted the limitations of HbA1c as a standalone marker of glycemic control in diabetes mellitus. Furthermore, recent comparative studies utilizing continuous glucose monitoring have shown that intrinsic factors, such as individual variability in erythrocyte lifespan [9], the presence of hemoglobin variants [10], and differences in glycation efficiency [11], may influence HbA1c values, resulting in either overestimation or underestimation of actual glycemic status. In hemolytic anemia, the shortened erythrocyte lifespan is known to cause falsely low HbA1c levels [12]. Therefore, a comprehensive differential diagnosis should be based on clinical symptoms and laboratory findings.

In a 2020 study, Carson et al. reported that among 14,099 individuals aged ≥20 years without diabetes who participated in the National Health and Nutrition Examination Survey III in the United States, an HbA1c level of <4.0% was observed in 97 patients (0.69%) [13]. Similarly, in 2004, Pettitt et al. identified an HbA1c level below 4.0% in 15 of 400 children without diabetes aged 11−14 years (3.75%) in the United States [14]. Although the prevalence of underlying conditions associated with low HbA1c levels varies by race and ethnicity [15], these findings suggest that such low levels are consistently detected at a low but notable frequency during population-based screening.

As alternatives to HbA1c for assessing glucose tolerance, two blood-based markers are commonly available: glycoalbumin and 1,5-anhydroglucitol (1,5-AG). Glycoalbumin reflects average glucose concentrations over the preceding two to three weeks and is not affected by erythrocyte lifespan; however, it may yield falsely elevated values in conditions associated with impaired serum albumin metabolism. Similarly, 1,5-AG reflects glycemic status over the past two to three weeks, but its levels are influenced by changes in renal function [16]. In our case, glycoalbumin was used as an alternative marker for glucose monitoring, demonstrating normal levels. Each marker possesses distinct characteristics and limitations, necessitating careful consideration when selecting the appropriate parameter for glycemic assessment.

## Conclusions

In pediatric populations, HbA1c measurements are infrequently performed. However, like adults, pediatric patients with hemolytic anemia experience a reduced erythrocyte lifespan, leading to spuriously lower HbA1c levels.

This case report highlights the importance of considering hemolytic anemia, including HS, when unexpectedly low HbA1c values are observed in pediatric patients, particularly those undergoing GH therapy. In such cases, glycoalbumin may serve as a reliable alternative marker for assessing glucose metabolism.
